# Role of Platelets in Leukocyte Recruitment and Resolution of Inflammation

**DOI:** 10.3389/fimmu.2018.02712

**Published:** 2018-11-20

**Authors:** Jan Rossaint, Andreas Margraf, Alexander Zarbock

**Affiliations:** ^1^Department of Anesthesiology, Intensive Care and Pain Medicine, University Hospital Münster, Münster, Germany; ^2^Interdisciplinary Centre for Clinical Research, University Hospital Münster, Münster, Germany

**Keywords:** platelets, leukocytes, neutrophils, inflammation, resolution

## Abstract

Platelets are most often recognized for their crucial role in the control of acute hemorrhage. However, current research has greatly expanded the appreciation of platelets beyond their contribution to primary hemostasis, indicating that platelets also actively participate in leukocyte recruitment and the regulation of the host defense in response to exogenous pathogens and sterile injury. Early recruitment of leukocytes, especially neutrophils, is the evolutionary stronghold of the innate immune response to successfully control exogenous infections. Platelets have been shown to physically interact with different leukocyte subsets during inflammatory processes. This interaction holds far-reaching implications for the leukocyte recruitment into peripheral tissues as well as the regulation of leukocyte cell autonomous functions, including the formation and liberation of neutrophil extracellular traps. These functions critically depend on the interaction of platelets with leukocytes. The host immune response and leukocyte recruitment must be tightly regulated to avoid excessive tissue and organ damage and to avoid chronification of inflammation. Thus, platelet-leukocyte interactions and the resulting leukocyte activation and recruitment also underlies tight regulation by several inherited feedback mechanisms to limit the extend of vascular inflammation and to protect the host from collateral damage caused by overshooting immune system activation. After the acute inflammatory phase has been overcome the host defense response must eventually be terminated to allow for resolution from inflammation and restoration of tissue and organ function. Besides their essential role for leukocyte recruitment and the initiation and propagation of vascular inflammation, platelets have lately also been implicated in the resolution process. Here, their contribution to phagocyte clearance, T cell recruitment and macrophage reprogramming is also of outmost importance. This review will focus on the role of platelets in leukocyte recruitment during the initiation of the host defense and we will also discuss the participation of platelets in the resolution process after acute inflammation.

## Introduction

The adequate regulated recruitment of leukocytes is an indispensable element of the innate immune response (1–3). Neutrophils are the predominant leukocyte subset that is recruited to inflamed tissue by the initial innate immune system response during the onset of inflammation. Their primary function lies in combating and removal of invading pathogens. If defective, reduced neutrophil recruitment and activation can be the cause of severe immune deficiency syndromes (4). Platelets are traditionally well recognized for their important role in primary hemostasis, yet research over the past decade has created a broader understanding of platelets as an essential element of the innate immune system (5, 6). Platelets serve as a major contributor of several pro-inflammatory chemokines and possess a whole inventory of surface receptors and adhesion molecules that enable platelets to bind to leukocytes as well as circulating pathogens, e.g., bacteria (7). Platelets circulate in the blood in a resting, quiescent state under physiological conditions. When platelets are activated, e.g., in the situation of acute vascular inflammation, they may physically directly interact with circulating leukocytes in the blood stream (8–13). The consequences of this interaction are manifold and include leukocyte activation and may enable leukocytes to fulfill their multiple cell-intrinsic functions and immunological task. Furthermore, the interaction of platelets in particular with neutrophils is a prerequisite for neutrophil extravasation and recruitment into inflamed organs in multiple inflammatory scenarios (14). Activated neutrophils can produce and release neutrophil extracellular traps (NETs) (15). NETs are capable of physically entrapping and killing circulating pathogens, e.g., bacteria. The interaction of platelets and neutrophils has been demonstrated to be a prerequisite for NET formation and release under different inflammatory conditions (9, 11, 12, 15, 16).

Beyond their role as auxiliary cells interacting with leukocytes and supporting them in fulfilling their immunological fate, platelets are also capable of direct interaction with circulating pathogens (7). The liver plays a central role in this process. Here, platelets patrol the microvasculature and perform multiple “touch-and-go” maneuvers with sinusoidal Kupffer cells. This interaction is mainly mediated by bond formation between GPIb on platelets and vWF expressed on hepatic Kupffer cells as an element of the innate immune surveillance system of the liver (17). Kupffer cells in the liver act like tissue-resident macrophages and may catch pathogens in the circulation that may have reached the bloodstream, e.g., from the intestines. This process changes platelet behavior in the liver and the interaction of platelets with Kupffer cells becomes permanent with subsequent activation of platelets. Therefore, the activated platelets may initiate the recruitment of circulation neutrophils to eliminate the entrapped pathogens. In this situation, platelets also serve as sentinel cells together with Kupffer cells to guide the innate immune responses elicited by leukocytes. Currently this phenomenon is best described in the liver (18). If platelets may also patrol other cell types apart from Kupffer cells in organs apart from the liver still has to be investigated.

The focus of this review is the role of platelets in leukocyte recruitment during inflammatory processes and during resolution from inflammation. We will emphasize the molecular mechanism regulating the complex formation between platelets and leukocytes and will highlight the functional consequences associated with these processes under different inflammatory conditions.

### Platelet physiology

Platelets do not possess a cellular nucleus and are essentially produced by fragmentation of megakaryocytes in the bone marrow, from where they are released into the circulation in large amounts. They are traditionally well known for their essential functions in primary hemostasis. Subendothelial structures of the extracellular matrix, e.g., collagen fibers and von-Willebrand factor, are usually inaccessible for circulating platelets. If the vessel wall injury leads to exposure of these molecules, platelet adhesion is triggered. The establishment of bonds between adhesion receptors on the cell surface of platelets and their ligands in the exposed extracellular matrix leads to signaling events in platelets and the cells become activated. Consequently, further adhesion molecules on platelets, e.g., integrins, become activated and platelets may release the contents of their intracellular granules including highly active pro-coagulatory mediators (e.g., ADP, thrombin and prostaglandins). The adhesion and activation of single platelets quickly recruits and activates further platelets to the site of vascular injury and leads to the formation of a leak-sealing thrombus. In addition to platelets, leukocytes are recruited and red blood cells are incorporated into the thrombus (19, 20). Apart from stopping blood loss from the injured vessel during traumatic tissue injury, a second objective is to limit and control the possible entry of exogenous pathogens, e.g., bacteria, via the wound surface into the circulation and thus into the organism. This process could potentially lead to local and/or systemically disseminated infections. From this perspective, the formation of an occlusive platelet thrombus resembles not only a barrier preventing blood loss to the outside, but may also serve as a shield to reduce local blood flow in the injured vessel and prevent the dissemination of pathogens from the outside into the organism, which may explain why platelets have evolutionary evolved to a cell type that also executes immunological functions.

A common cell line known as “hematocytes” were abundant in certain invertebrates and very early vertebrates, and are still conserved e.g., in horshoe crabs, a member of the anthropod family which originated about 450 million years ago. Hematocytes combined immunological as well as hemostatic functions primarily found in leukocytes and platelets of today's mammals (7, 21, 22). During evolution and the appearance of mammals, several more specialized hematopoietic cell lines originated from hematocytes, including lymphocytes, monocytes, neutrophils, and eventually also platelets. These cell lines are characterized by the fact that they are actually able to execute less cell-autonomous functions, but with higher specialization. As a matter of fact, the relationship between the hemostatic and the immune system remained very tight during the development of higher organism with a high degree of interconnectivity. The term “immunothrombosis” has lately been proposed to describe the pathophysiological events modulated by immune cells in cooperation with the coagulation system to facilitate the recognition, containment and destruction of exogenous pathogens during vascular inflammation (23).

Platelets possess a wide inventory of cellular adhesion molecules. These molecules have individual functions and enable platelets to act in different hemostatic and inflammatory situations. Furthermore, they contain intracellular granules (α-granules, dense granules and lysosome granules) packed with various pro-coagulant and immune-modulatory mediators that may be released in response to exposure to different activating stimuli (24). Platelets circulate in the blood stream in very high numbers and it does not come as a surprise that the immune system utilizes platelets to serve as cellular sentinels which are needed for broad surveillance of the circulation and detection of pathogens and possible threats.

### Platelet cellular activation, adhesion molecules and surface receptors

Initial platelet activation is the key element in platelet function. Platelets can be activated either by binding of soluble platelet agonists, e.g., ADP or thrombin, or by exposure to subendothelial extracellular matrix components, e.g., collagen (25, 26). Ligand binding to platelets leads to the activation of intracellular signaling pathways which cause platelet shape change and cytoskeletal rearrangement, release of platelet granule content and the activation of cell surface adhesion molecules. Platelet granules are a source of many pro-inflammatory and pro-coagulant mediators. These also include chemoattractive cytokines and chemokines and allow platelets to actively fulfill their role in in primary hemostasis and also in inflammatory processes. Interestingly, experimental evidence suggests that the response of platelets to different activating stimuli is actually not uniform. This indicates the existence of a stimulus-dependent platelet response, e.g., degranulation (27, 28). Further *in vitro* studies revealed more insights of stimulus-dependent release characteristics of platelet granule content. It could be shown that although the composition of released platelet granule-derived mediators following agonist exposition appeared to be mixed in a stochastic manner, the temporal kinetics of platelet granule release clearly followed different stimulus-characteristic patterns (29). These findings are supported by imaging studies using immunofluorescence staining to visualize pair-wise packing of different molecules stored in α-granules. Here, the packaging pattern of platelet granule content also followed a stochastic distribution (30). However, alternative mechanism other that individual platelet granule packing and release might contribute to a stimulus-dependent platelet response, e.g., incomplete granule fusion upon content release and the interaction of individual mediators in a complete signaling network, and this questions remains the topic of current investigations.

Apart from activation by soluble mediators of cellular interaction by direct binding of ligands for platelet cell surface adhesion molecules, platelets are capable of direct interactions with bacteria. For example, platelets may bind and take up Listeria monocytogenes, a facultative intracellular bacterium. In turn, platelets selectively bind to DCs (CD8α^+^ dendritic cells) for pathogen delivery and presentation initiating an adaptive immune response (31). In another example, platelets have been shown to be necessary for viral clearance by cytotoxic T cells in lymphocytic choriomeningitis virus (LCMV) infections (32, 33). These findings also underline the fact that platelets do not only play a crucial immunological role by interaction with the innate immune system but are also capable of directly affecting the adaptive immune response.

To acknowledge the exact role of platelets in initiating and modulating the immune response to inflammatory stimuli, the function of the main platelet surface adhesion molecules and platelet surface receptors are of great importance. Integrins are a family of surface adhesion molecules which are abundantly expressed an many cell types where they mostly mediate direct cell-matrix and cell-cell interactions (34). Platelets express several integrins, which are the most important class of cell adhesion molecules on platelets. Integrins are formed as a heterodimer consisting of an α- and a β-chain. Integrins reside in an inactive state not capable for ligand binding under resting conditions (low affinity conformation). If activated, conformational change of both the α- and β-subunit occurs and access to the ligand binding site is granted (high affinity conformation) (35). Individual integrins may also possess the ability to change into an intermediate conformation with limited ligand binding affinity. Platelets express mostly integrins of the β_1_- and β_3_-subfamily, including α_IIb_β_3_ (GPIIb/IIIa), α_2_β_1_ (VLA-2, GPIa/IIa), α_5_β_1_ (VLA-5), and α_6_β_1_ (VLA-6) (36, 37). Platelet integrins fulfill divergent functions in the interplay of platelets with the subendothelial, extracellular matrix, leukocytes and endothelial cells (36, 37). A second important feature of integrins is their ability to transduce activating signals into the cell in a process called outside-in signaling (38). Thus, following ligand binding to integrins an intracellular signaling cascaded may be triggered inside platelets leading to further cell activation or degranulation.

**Figure 1:**
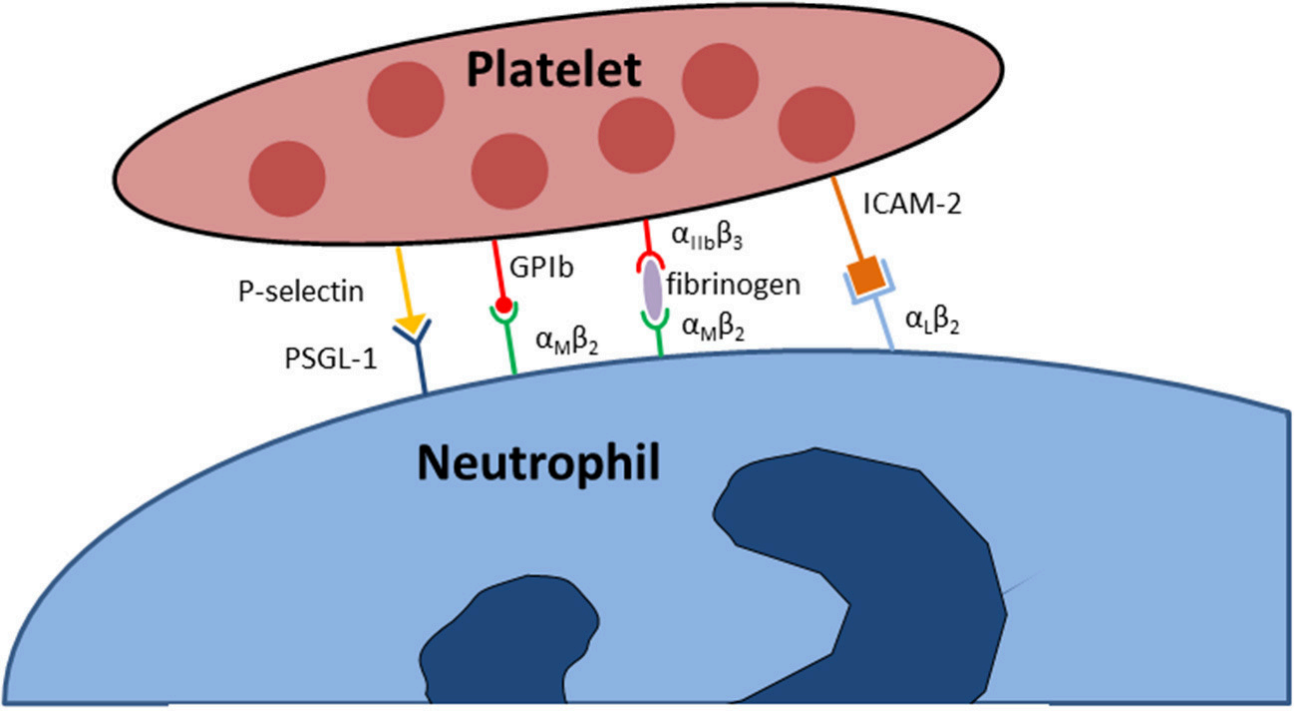
Adhesion molecules implicated in the physical interaction between neutrophils and platelets.

Furthermore, platelets express additional glycoprotein complexes, including the glycoprotein (GP) Ib-V-IX complex. This molecule cluster serves as the most important binding partner on platelets for von Willebrand factor (vWF) (39). This complex generally mediates the first contact of platelets with structures of the subendothelial, extracellular matrix which is exposed following blood vessel injury. Another important glycoprotein, GPVI, can bind to collagen. Noticeably, platelet activation also leads to an increased surface expression and activation of glycoproteins. The glycoprotein GPIIbIIIa, which is a synonym for the integrin α_IIb_β_3_ is a binding partner for fibronectin, retronectin and vWF (36, 40). GPIIbIIIa is the most abundantly expressed platelet surface adhesion molecule and in its activated conformation binds various ligands, e.g., fibrinogen, vitronectin, fibronectin, vWF or thrombospondin (40). Platelet adhesion molecules are involved in many immunological tasks elicited by platelets. *In vitro* studies under static conditions demonstrated that β_3_-integrins on platelets are necessary to mediate firm platelet adhesion to the cell surface of inflamed endothelial cells (41, 42) and both the used of blocking antibodies directed against the integrin α_IIb_β_3_ on platelets and the genomic knockout of this adhesion molecule caused less platelet adhesion to inflamed endothelial cells *in vivo* (43). Besides this very important role in mediating the contact to endothelial cells the integrin α_IIb_β_3_ is also crucially involved in the initiation and regulation of direct physical interactions of platelets with leukocytes under inflammatory conditions. Here, the integrin α_IIb_β_3_ serves as a binding partner the integrin α_L_β_2_ (Mac-1) on neutrophils via a bridge of soluble fibrinogen (Figure 1) (40). Beyond physical bond formation, the binding of platelet α_IIb_β_3_ to neutrophil Mac-1 also initiates outside-in signaling into neutrophils and is necessary for NET formation and leukocyte recruitment (12, 16).

Selectins are adhesion molecular that are abundant on numerous cells types, including endothelial cells, leukocytes, and platelets (44). P-selectin is stored in platelet α-granules in resting platelets. When activated, platelets incorporated P-selectin into the plasma membrane where it becomes available for interaction with its binding partners, e.g., P-selectin glycoprotein ligand-1 (PSGL-1) on neutrophils and monocytes (Figure 1) (7, 45–49). Experimental evidence suggests that the binding of PSGL-1 to P-selectin is necessary to initiate the first interaction between platelets and neutrophils (50, 51). Further adhesion molecules expressed on platelets include several cellular adhesion molecules, including the junctional adhesion molecules (JAM-A, JAM-C), intercellular adhesion molecule (ICAM)-2, and PECAM-1 (platelet endothelial cell adhesion molecule-1) (37). In particular, ICAM-2 on platelets is capable of binding to Mac-1 on neutrophils, but the physiological relevance of these adhesion molecules for the immunological functions of platelets and neutrophils are not fully understood.

Besides adhesion molecules platelets also express different receptors on their cell surface, e.g., complement receptors, pattern recognition receptors (PRRs) of the Toll-like receptor family (TLR1-9) and receptors for detecting immunoglobulins (FcR) (7, 52, 53). These receptors provide platelets with the ability to sense and respond to endogenous pro-coagulant and/or pro-inflammatory mediators, exogenous pathogens and incorporate these signals into cell activation (52). TLRs are a family of evolutionary highly conserved pattern recognition receptors to sense common motifs of exogenous pathogens, termed “pathogen associated molecular patterns” (PAMPs). The detection of PAMPs by TLRs leads to the initiation of an adequate immunological response (54). Functional TLR4 is expressed on platelets (52) and its main function is to recognize lipopolysaccharide (LPS) (55). However, TLR4 binding of LPS does not directly lead to platelet activation and aggregation (52, 56), but merely causes significant platelet priming in the lung and in the liver of mice during LPS-induced vascular inflammation (55). The reasons for this phenomenon are not fully understood to date, but it has been proposed that LPS-induced platelet priming induced the increased production of the pro-inflammatory cytokine TNF-α in the context of bacteremia, and the LPS-binding to TLR4 leads to increased phagocytosis by mononuclear cells (57). Furthermore, it is known that TLR4 activation during systemic inflammation causes the production and release of neutrophil extracellular traps (NETs) helping to catch circulating pathogens from the bloodstream (9). The exact direct or indirect molecular interactions between neutrophils and platelets following platelet TLR4 activation remain poorly defined.

**Figure 2:**
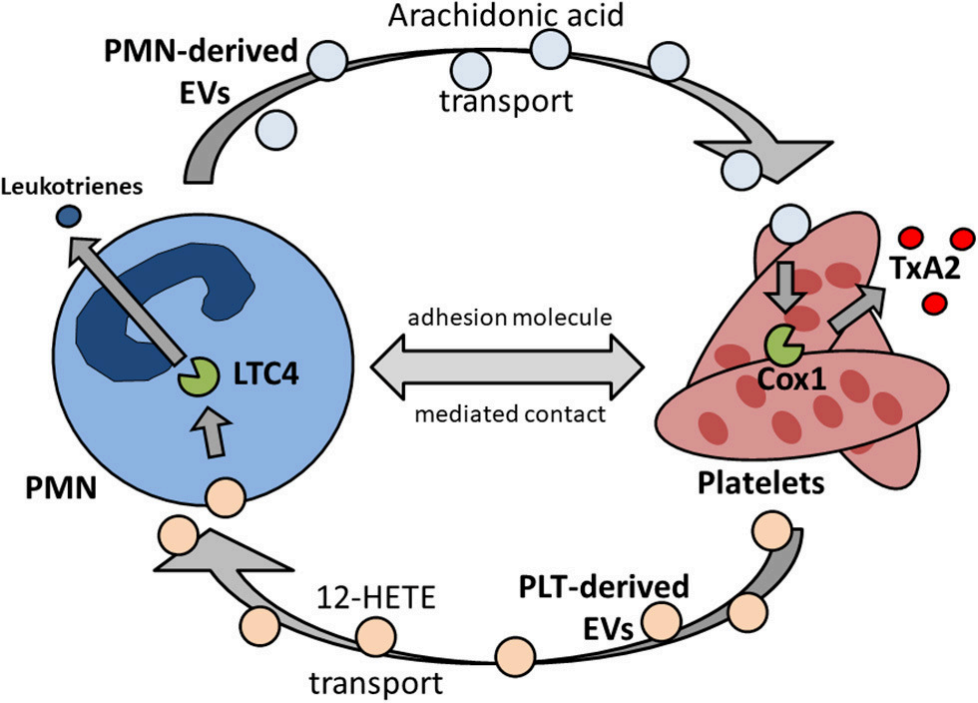
Reciprocal transcellular exchange of substrate and metabolites by extracellular vesicles governs synthesis and release of pro-inflammatory mediators. PMN, polymorphonuclear granulocyte, neutrophil; EV, extracellular vesicles; Cox1, cyclooxygenase 1; TxA_2_, thromboxane A_2_; PLT, platelet; 12-HETE, 12-hydroxyeicosatetenoic acid; LTC4, leukotriene 4 synthase.

Platelets express various prostaglandin receptors. Prostaglandins are synthesized from membrane-derived phospholipids and are involved in the modulation and regulation of a wide range of physiological processes, e.g., in the cardiovascular, central nervous and immune system. Platelets possess receptors to sense thromboxane, prostacyclin (PGI_2_), PGD_2_, and PGE_2_. Thromboxane A_2_/prostaglandin H_2_ (TxA_2_/PGH_2_) receptor activation causes the activation of phospholipase A_2_. This in terms leads to the amplification of platelet activation by autocrine mechanisms. The major inhibitory prostaglandin receptor on platelets are prostacyclin (PGI_2_) receptors. Prostacyclin is synthesized and released by resting, non-inflamed endothelial cells. PGI_2_ receptors on platelets sense prostacyclin and suppress platelet activation (58). TxA_2_ has been shown to be an major regulator of inflammatory processes *in vivo* and is involved in endothelial cells activation and amplification of inflammation (8, 13, 59). Platelets may synthesize TxA_2_ by cyclooxygenases, but they are lacking sufficient substrates on their own (60). The main substrate needed for prostaglandin synthesis is arachidonic acid, which is produced by phospholipase A_2_ by enzymatic hydrolysis of plasma membrane phospholipids. TxA_2_ production in platelets is significantly increased in the presence of neutrophils. Here, arachidonic acid is shuttled from neutrophils by transcellular metabolism into platelets (13, 61). This process substantially increases platelet TxA_2_ production. Of note, the binding of P-selectin on platelet to PSGL-1 on neutrophils plays a role in this process, possibly by providing and maintaining the physical proximity between the two cell types (13, 62) (Figure 2).

### Platelet-derived soluble mediators

Platelet granules are a storage for various mediators. These include pro-coagulant factors, e.g., ADP and mediators without a direct hemostatic function, e.g., PDGF (platelet-derived growth factor). PDGF plays a role in the regulation of wound healing during and after local inflammation (63). Beyond pro-coagulant factors, platelet granules are also a packed with different pro- and anti-inflammatory mediators, e.g.. transforming growth factor-β (TGF-β) (64). TGF-β, together with IL-10, is one of the most important negative regulatory chemokines of inflammatory processes. Platelets are a major source of TGF-β in the organism, and ITP (idiopathic immune thrombocytopenia) in humans is also characterized by reduced plasma TGF-β levels, which may rise again if low platelet counts during ITP recover to normal values (65, 66). Platelets are also a major source of the chemokines CXCL4 (platelet factor 4), CCL5 (RANTES), and CXCL7 (Neutrophil-activating peptide-2, NAP-2) which may be liberated by activated platelets and are actively involved in neutrophil recruitment and activation (67). Platelet CXCL4 and CCL5 have been shown to be crucial involved in the recruitment and activation of neutrophils during the pathogenesis of acute lung injury (12, 68). Here the two chemokines are deposited on the luminal cell surface of endothelial cells to form a heterodimer and are needed to induce endothelial arrest of intravascular neutrophils (68–71). Furthermore, platelet CXCL4/CCL5 heterodimer binding to neutrophils has been shown to be necessary for neutrophil NET formation and release during the pathogenesis of acute lung injury (12). CXCL7 (NAP7) is a potent CXCR2 agonist and is produced from its precursor molecules CTAP (connective tissue-activating peptide)-III and PBP (platelet basic protein). CXCL7 has also been implicated in the complex formation of neutrophils and platelets under inflammatory conditions *in vitro* and *in vivo* (72) and promotes chemotaxis of neutrophils (73). CXCL7 and CXCL4 possess unique structural properties that modulate neutrophil recruitment and processes including chemokine heterodimer formation, glycosaminoglycan (GAG) interactions, and gradient formation. CXCL7 is known to form several biologically active heterodimers with other chemokines, e.g., CXCL7-CXCL1, CXCL7-CXCL4, and CXCL7-CXCL8 heterodimers (74). Interestingly, the binding properties of these heterodimers to GAGs on endothelial cells are substantially different compared to monomeric CXCL7 and this also modulated the receptor binding of the chemokines (74, 75). It was described that the binding of GAGs to monomeric CXCL7 might dynamically modulate the chemokines receptor binding properties and that the GAG-bound monomeric CXCL7 shows less receptor binding affinity than GAG-bound CXCL7 heterodimers (75). CXCL7 liberation by platelet degranulation, interaction with GAGs on the endothelial surface and the resulting gradient formation between free and GAG-bound forms of CXCL7 complexes are also events that contribute to the directed neutrophil recruitment to the site of vascular inflammation (76). CXCL7 also forms tetramers, but to this date nothing is known about the pathophysiological role of these complexes. Interestingly, a negative feedback loop exits to limit the pro-inflammatory action of CXCL7 by CTAP-II (the precursor molecule of CXCL7) inducing the downregulation of CXCR2 on neutrophils. Likewise, also PBP may dampen CXCL7-induced neutrophil activation, degranulation and chemotaxis (77). These studies provide evidence that the release of platelet-derived chemokines may itself lead to the desensitization of chemokine receptors on neutrophils. This may represent an important negative-feedback regulation limiting neutrophil activation.

Platelets are not only a source of chemokines, but the cells themselves also possess chemokine receptors and respond to chemokine stimulation (78). The most prominent chemokine receptors expressed on the cell surface of platelets include CXCR4, CX3CR1, CCR1, CCR3, and CCR4 (79, 80). Several chemokines are known as binding ligands of these receptors and induce platelet activation, including SDF-1 (CXCL12) released by inflamed endothelial cells, TARC (CCL17) and MCD (CCL22) which may be produced by mononuclear cells (81). Besides platelet shape change and activation of cell adhesion molecules, another major consequence of platelet activation by chemokines is the platelet degranulation during which platelet P-selectin stored in platelet granules is integrated into the platelet plasma membrane. The binding of P-selectin of activated platelets to PSGL-1 on neutrophils is an essential step during the formation of physical platelet-neutrophil interactions during inflammatory processes. The formation of platelet-neutrophil complexes is of great importance for neutrophil recruitment and neutrophil function during the pathogenesis of numerous inflammatory diseases (see paragraph below). Current research has demonstrated that platelets are capable of active migration, and activation of CXCR4 on platelets by CXCL12 is critically involved in this process (82–84), but the pathophysiological relevance of this finding during inflammatory diseases has yet to be investigated.

### Adenosine diphosphate receptors

Adenosine diphosphate (ADP) is a platelet agonist and kept in dense platelet granules in the quiescent state. Upon platelet activation, ADP is set free following platelet degranulation. Extracellular ADP may bind to P_2_Y_1_ an P_2_Y_12_ receptors. Both receptors are GTP-coupled, platelet-activating receptors. Furthermore, APD may also act by binding to the receptor P_2_X_1_, which acts as an ion channel for free calcium ions upon ligand binding and subsequently leads to platelet cytoskeletal rearrangement and induction of platelet shape change. ADP alone is a rather weak platelet agonist. However, it significantly increases the platelet-activating response induced by additional platelet agonists, e.g. thrombin, and leads to the synthesis and liberation of TxA_2_ from activated platelets, which in turn resembles a strong paracrine platelet agonist. Furthermore, ADP binding to platelets induces the platelet integrin activation (e.g., GPIIbIIIa, integrin α_IIb_β_3_) and platelet aggregation (85). Platelet activation by ADP plays an important pathophysiological role during several inflammatory diseases, including sepsis (86–89).

### Platelet-leukocyte interactions

Direct physical interactions between platelets and leukocytes are regulated by several distinct molecular interactions but are also enforced by non-biological physical propensities of the vascular system. This is in parts explained by the rheological properties of blood as a mixture of fluids and corpuscular components (90, 91). Here, erythrocytes and larger cells (e.g., leukocytes) stay relatively centered in the middle of the blood flow, whereas platelets are more enriched in the peripheral vicinity of the blood flow closer to the endothelial cell surface lining the inner lumen of the blood vessel. The enrichment of platelets near the vessel wall make encounters with leukocytes in this area more likely. This increases the chance of transient platelet-leukocyte interactions in this area, which might become permanent in case of vascular inflammation with activation of platelets, leukocytes and endothelial cells (91). But also under physiological condition in the absence of inflammation, a small number of transient platelet-neutrophil interactions has been described close to the vascular endothelium (92, 93). The first and probably most important physical interaction between platelets and leukocytes, in particular neutrophils, is established by bond formation between P-selectin on activated platelets and PSGL-1 constitutively expressed on neutrophils (Figure 1). This causes the phenomenon of secondary capturing of free-flowing neutrophils by initial binding of PSGL-1 on these cells to P-selectin expressed by adherent, activated platelets on the vascular endothelial cell surface (94). However, ligand binding to neutrophil PSGL-1 does not only mediate cell-cell interactions, but also induces intracellular signaling. Following PSGL-1 engagement, a cascade of signaling events in neutrophils, including BTK (bruton's tyrosine kinase), Src and MAP kinases, leads to the activation of integrins expressed on neutrophils, e.g., α_L_β_2_ (LFA-1) and α_M_β_2_ (Mac-1) (50, 95–100). LFA-1 is a binding partner of ICAM-2 (intercellular adhesion molecule 2) on platelets, although the exact pathophysiological contribution of this interaction under different inflammatory conditions remains unclear (101–103). Activated Mac-1 on neutrophils is of particular importance for the interaction of neutrophils with platelets, as it is a direct binding ligand for the platelet surface adhesion molecule GPIbα and also indirectly binds to activated platelet GPIIbIIIa through a “bridge” of fibrinogen (Figure 1) (104, 105). The role of GPIIbIIIa binding to Mac-1 in the regulation of neutrophil recruitment and activation has been shown in different inflammatory diseases, e.g., pulmonary inflammation, whereas GPIbα binding to Mac-1 is known to regulate platelet adherence *in vitro* and is involved in leukocyte recruitment following femoral artery injury in the murine system (105–107). While research over the past decades has revealed several mechanisms by which the platelet and neutrophil may directly and indirectly interact, we are just beginning to understand the specific role and contribution of this phenomenon in different inflammatory diseases. While some diseases models appear to be critically dependent on this interaction, others may not, and even within the same organ system differences may exist in between different inflammatory stimuli (3, 8, 12).

Beyond mediating physical cell-cell interactions, the binding of platelets to neutrophils also modulates and induces cellular immunological functions in neutrophils. A major cellular function of neutrophils is the production and release of ROS (reactive oxygen species). Due to their nature as free radicals, ROS are extremely cell-toxic, and they aid in the destruction of pathogens, e.g., invading bacteria at sites of infection. The complex formation and interaction of platelets and neutrophils induces subsequent integrin-mediated outside-in signaling into neutrophils, which in addition to chemokine stimulation triggers ROS production and release by activated neutrophils. It has been shown that platelet binding to neutrophils increases neutrophil ROS generation efficiency (108, 109). The molecular interaction of P-selectin on platelets and neutrophil PSGL-1 is also of great importance for this process *in vitro* and *in vivo* (110, 111). Likewise, pharmacological blockade of ADP binding to its cellular platelet receptor P_2_Y_12_ also impaired ROS production in neutrophils (112). As platelet activation by ADP also induces P-selectin mobilization and membrane integration, an implication of the P-selectin/PSGL-1 binding system could be involved in the underlying molecular mechanism. However, exact evidence for this hypothesis is lacking as ADP stimulation of platelets also induces the activation of additional platelet surface adhesion molecules. The second important immunological function by which neutrophils eliminate pathogens is phagocytosis and this process is also affected by platelet-neutrophil interactions. Here, indirect interaction pathways mediated by soluble inflammatory mediators, e.g., prostaglandins and purine nucleotides, play a more important role than direct ligand-receptor interactions (113–116). However, direct cellular interactions also seem to be involved, at least under distinct inflammatory conditions. This was shown by results from study utilizing a periodontitis model where efficient phagocytosis by neutrophils relied on the complex formation of neutrophils and platelets (117), indicating a possible tissue- and stimulus-specificity of platelet-dependency.

The third cell-autonomous immunological feature by which neutrophils may directly engage and kill bacteria is the formation and released of neutrophil extracellular traps (NETs) generated by “NETosis” (15). NETs are essentially decondensed nuclear chromatin, which is decorate with granular proteins from neutrophils and spun into the extracellular space. Although the generation of NETs leaves the neutrophils without a nucleus, the cells are still alive and are capable or cellular functions, e.g. intravascular crawling and transmigration (118). Physically, NETs may act like real-life fishing nets and entangle pathogens circulating in the blood stream. The relevance of this effect has been shown in different models of inflammatory diseases involving the blood-borne distribution of pathogens in the organism (11). Interestingly, NETs are also implicated in the pathogenesis of inflammatory disorders not involving infectious pathogens or stimuli. Here, NET formation has been shown to be a prerequisite for efficient neutrophil recruitment from the vasculature to the site of inflammation and platelet-neutrophil complex formation has been demonstrated to be critically involved in this process (12, 16). The pattern recognition receptor TLR4 is expressed on platelets and may be activated by binding of bacterial products, e.g., LPS. TLR4 activation on platelets leads to neutrophil NET formation and liberation, but the exact mechanism remains unclear (9). Furthermore, the direct physical interaction of platelets and neutrophils by binding of activated GPIIbIIIa to Mac-1 on neutrophils (via a bridge of fibrinogen) also induces NET formation by neutrophils together with simultaneous activation of GPCRs (G-protein coupled receptors) on neutrophils by CXCL4/CCL5 heterodimers released by activated platelets to facilitate neutrophil recruitment during sterile pulmonary inflammation (12). An example for indirect platelet-neutrophil interactions inducing NET release is hBD-1 (human β-defensin 1). Platelets secrete hBD-1 in response to contact with toxin from S. aureus, and hBD-1 has been shown to cause NET release by neutrophils (119).

### Platelet microparticles

Although platelets are small fragments originating from larger cells (megakaryocytes) themselves, they are still capable to generate microparticles with dimensions in the sub-micrometer range (120). Current research has demonstrated that microparticles are associated with multiple physiological and pathophysiological functions (121). Also not restricted to platelets as originating cells, the majority of microparticles in the blood are actually coming from platelets (122). The fact that microparticles may carry certain proteins that are normally not expressed or only expressed in much smaller quantities in their originating cells indicates that microparticles are produced and packed with dedicated proteins in an active process and not just by random cell sequestration (123). Yet the exact regulatory processes guiding these pathways in platelets still have to be investigated. Microparticles may also well interact with and bind to leukocytes, since they inherit the adhesion receptors, e.g., P-selectin and the platelet integrin α_IIb_β_3_, from the platelets cells surface (124–126). However, it is unknown if the platelet integrin α_IIb_β_3_ is activated on the microparticle surface and contributes to adhesion. Microparticles originating from platelets are also capable of binding to other cell types than leukocytes, e.g., endothelial cells. In fact, excessive microparticle binding to the surface of endothelial cells may lead to endothelial cell activation (127).

As a specialized class of microparticles, extracellular vesicles (EVs) are actively released by cells, e.g., neutrophils, following active cell-internal production, packaging and release. EV transport in between neutrophils and platelets has gained attention as it could be demonstrated that intermediate metabolites necessary for sufficient prostaglandin synthesis and release by platelets are shuffled from neutrophils into platelets via specific EV release and uptake (13, 61). Likewise, the transcellular transport vice versa from platelets to neutrophils also plays an important pathophysiological role. Here, neutrophils receive 12-hydroxyeicosatetenoic acid (12-HETE) from platelets to synthesize leukotrienes (128). Interestingly, this interaction also modulates LTC4 synthase activity further downstream in neutrophils, as does the transport *vice versa* from neutrophils to platelets regulate cyclooxygenase 1 activity in platelets (62, 129). It seems fair to argue that transcellular metabolite exchange between neutrophils and platelets via EVs is a two-way interaction (Figure 2) (130).

### The role of platelets in the pathogenesis of acute inflammatory diseases

Acute lung injury is a respiratory disorder characterized by pulmonary leukocyte recruitment and edema formation leading to impaired gas exchange with severe consequences for patients, depending on its severity (131, 132). It may occur in response to different stimuli, e.g., pulmonary bacterial infections, sepsis, and aspiration of gastric content (133). The pathogenesis of pulmonary inflammation and acute lung injury has been demonstrated to rely on platelet-neutrophil complex formation in various disease models. They include transfusion-related acute lung injury (TRALI) (10, 16, 134), LPS-induced lung injury (68, 135), acid-induced lung injury (8), and ventilator-induced lung injury (VILI) (12). Experimental evidence has demonstrated that platelet-neutrophil complexes can be detected in a circulating manner in the blood as well as directly attached to the vessel walls in the lung microcirculation as early as 30 min after exposure to the inflammatory stimulus (8). Here, complex formation involving platelet P-selectin is critically involved and pharmacological blockade of this molecule or cellular depletion of platelets showed a protective effect in reducing immune cell recruitment and limiting the vascular permeability increase. Platelet-neutrophil complex formation also regulated the production of TxA_2_ and this caused endothelial cell activation and expression of the endothelial cell adhesion molecule ICAM-1 (8). The importance of the direct cellular interaction between platelets and neutrophils in this process was also underlined by a later study demonstrating that platelet-neutrophil complex formation is necessary for the transcellular transport of metabolites from neutrophils into platelets to booster TxA_2_ production during the host immune response following induction of bacterial pneumonia (13). Pulmonary inflammation may also be induced by non-inflammatory stimuli, e.g., barotrauma during ventilator-induced lung injury (VILI). The interaction of platelets and neutrophils has also been shown to be required for neutrophil recruitment into the lung during VILI by intravascular formation and release of NETs (12). Moreover, Grommes et al. could show that platelet-neutrophil complex formation is also involved in a murine model of LPS-induced lung injury (68).

Massive transfusion of blood products, e.g., during severe hemorrhage following trauma, may cause transfusion-related acute lung injury (TRALI) and is a feared complication in transfusion medicine (134). It has become evident, that the deterioration of gas exchange during TRALI is not a result of an intravascular fluid overload, but essentially involved immunological pathways leading to inflammatory activation of the pulmonary endothelium, immune cell recruitment and increased vascular permeability. Lately, the platelets and platelet-neutrophil interaction shave been shown to be critically involved in the development of TRALI (109). Here, platelets may liberate CD154 in response to TLR-ligand binding (136). Subsequent CD154 binding to CD40 on neutrophils may cause activation of these cells and lead to neutrophil recruitment into the lung (137).

Whether platelet depletion or the attenuation of a platelet-elicited immunological response is associated with improvement of deterioration of the outcome critically relies on the nature of the underlying inflammatory stimulus in a particular model. Whereas the attenuation of the innate host immune response may be beneficial in disease models using aseptic inflammatory stimuli (e.g., LPS inhalation, intratracheal acid instillation or TRALI), the same intervention may substantially worsen the outcome in an infectious model, e.g., following the intratracheal instillation of viable bacteria to induce pulmonary inflammation (13, 138). In addition, it remains unclear if and how platelets may reach other compartments in the lung than the intravascular space, e.g., the lung interstitium or the alveoli. Evidence from first studies suggest that platelets may also be present in the lung as far as in the alveoli (139). There is first evidence that platelets may also be released by megakaryocytes situated outside of the bone marrow, e.g., within the pulmonary microcirculation and it was claimed that this extra-medullar platelet synthesis contributes to a large amount of circulating platelets (140). Yet, the specific contribution to this putative new platelet reservoir in the lungs as well as the functional role of platelets in the different compartments of the lung and their specific contribution to the disease progression here still has to be investigated in more detail.

Platelets are traditionally thought to possess only little motile capabilities, mainly related to rolling, adhesion and aggregation. However, several recent reports have substantially challenged this dogma with the discovery of platelet migration. First reports indicated that human platelets adapt to the application of high shear forces by cellular polarization and flow-directed migration and show migratory behavior toward a SDF-1 (stromal cell-derived factor 1) gradient *in vitro* (82, 83). First *in vivo* studies demonstrated that platelet migrate into the extravascular compartment of the lung during allergen-induced airway inflammation (141). Lately, Gärtner et al. showed that platelet migration occurs under inflammatory conditions in mice *in vivo* and is crucial for bacterial host defense and bundling of bacteria for improved phagocytosis (84). For this process, GPIIb/IIIa, as well as ADP and thromboxane A_2_ are needed, and it has previously been shown that platelet-TxA_2_ contributes to the neutrophil recruitment into the lung (13). Interestingly, it was noted that platelets adherent to leukocytes migrate faster than independent platelets, and only a certain percentage of all platelets does migrate (84). However, the relevance of platelet migration in the lung remains unclear to this date.

The liver represents another organ in which platelets and platelet-neutrophil complexes are prominently involved in host defense. The liver is uniquely characterized by the fact that the hepatic microcirculation is placed second in line beyond the intestinal microcirculation, connected by the portal vein. Thus, the liver is also exposed as the first organ that might be passed by invading exogenous bacteria from the intestinal tract (142). The liver is equipped with a unique and specialized immune surveillance system that resides in the livers sinusoidal space where Kupffer cells sense distinct bacterial structures and components. Circulating platelets are in constant temporary contact with Kupffer cells, performing “touch-and-go” maneuvers. Once Kupffer cells become activated following pathogen contact, platelets permanently attach to Kupffer cells by GPIIb-mediated adhesion and attract neutrophils to the liver sinusoids to aid in pathogen clearance (17).

Overwhelming systemic inflammation may occur due to uncontrolled local inflammation and can be potentially life-threatening for host. Interestingly, systemic inflammation and sepsis are often accompanied by transient low platelet counts in the blood, which may rise again after the initial phase of systemic inflammation is overcome (143). One factor contributing to decreased blood platelets counts may be the occurrence of DIC (disseminated intravascular coagulation) consuming platelets. However, emerging evidence also hints to a possible consumption of platelets caused by immunological processes in the circulation during systemic inflammatory disorders (144). This is also supported by results from clinical studies indicating that low circulating platelet counts are often associated with increased circulating microparticles and that circulating platelets from patients with sepsis show increased P-selectin surface expression levels indicating platelet activation (126).

### Platelets in the resolution of inflammation

Timely resolution of inflammation is important to impede uncontrolled host tissue destruction and organ dysfunction leading to chronic inflammation and fibrosis (145). It is essential that neutrophils are rapidly and efficiently removed from the inflammatory site upon clearance of the invading microorganisms thus avoiding excessive tissue damage (146). Neutrophil apoptosis and consequent engulfment by macrophages is the major route by which the host clears neutrophils. Efficient phagocytosis of apoptotic neutrophils by macrophages not only prevents their secondary necrosis but also turns pro-inflammatory macrophages into cells with an anti-inflammatory, reparative signature (147). Dysfunction in the neutrophil apoptosis machinery is considered critical for the pathogenesis of many chronic human inflammatory diseases, e.g., pulmonary fibrosis after ARDS (148). While various pro-resolving mediators and pathways that govern resolution from inflammation in the lung have been described, the role of platelets in this process remains vaguely investigated. Interestingly, a current report also indicated that delayed neutrophil apoptosis and clearance are also associated with delayed recovery from ischemia/reperfusion-induced acute kidney injury and accelerated renal fibrosis (149).

In the lunge, several studies have provided evidence that platelets do not only act during vascular inflammation within the intravascular compartment, but eventually also appear in the lung alveoli. Platelets have been found to extravasate and accumulate beneath the airways in a model of allergic inflammation (141). Further evidence supported the observation that platelets, eventually coupled to leukocytes, can be detected in the bronchoalveolar lavage fluid after induction of pulmonary inflammation (139). Supporting this finding, platelets could also be found in the BAL of mice after intratracheal instillation of LPS (150). Platelets were long thought to be passive corpuscular blood components that reach their site of action by chance, enforced by their sheer numbers (151). Contradicting this dogma it could recently be demonstrated that platelets are capable of active migration (84). This observation may contribute to the concept that the distribution of platelets is not only restricted to the intravascular compartment, but that platelets also translocate toward the alveolar space, i.e., into the organ tissue. However, previous studies in other organs provided hints that platelets may not only be important for the propagation of vascular inflammation. It could be shown that platelet activating factor (PAF) plays a role in mediating the uptake of urate crystals during the resolution of gouty inflammation (152). Platelets are also a major source for anti-inflammatory mediators of the lipoxin family, e.g., specialized pro-resolving mediators (SPMs) such as resolvins and maresins (153). These lipoxins are produced and released already during the inflammatory onset phase of acute inflammation and their concentrations sharply rise during the convergence toward the resolution phase (154). Interestingly, these lipoxins also promote phagocytic clearance of apoptotic immune cells, e.g., neutrophils, during resolution (155).

Neutrophils and macrophages have traditionally been regarded as dominant cell types during the resolution of inflammation. Regulatory T cells (T_regs_) represent a T cell subpopulation with predominantly immune regulatory functions and are mainly acting immunosuppressive. T_regs_ are a source of the anti-inflammatory cytokines interleukin 10 (IL-10) and transforming growth factor ß (TGF-ß). Yet, it is unknown how exactly platelets, macrophages and T_regs_ participate in the resolution of pulmonary inflammation. Platelets have been previously described to interact with regulatory T cells under inflammatory conditions. In this context, it could be shown that platelets are needed to control the anti-inflammatory actions of CD4^+^ regulatory T cells following burn injury trauma in mice (156). In another organ, platelets have also been shown to interact with CD4^+^ T cells in the liver following ischemic injury and during atherosclerosis (157, 158). Interestingly, platelets are also thought to be capable of inducing CD4^+^ T cell differentiation by both the release of distinct chemokines and by direct cell-cell contact with T cells. As a consequence, IL-10 production and release by T cells was enhanced (159).

## Conclusion

While platelets are traditionally perceived as essential elements of primary hemostasis, the contemporary perception of their pathophysiological role should also clearly include their prominent contribution to inflammatory processes. Current and past research has shed light on their participation in the generation of an adequate immune response. Here, both the direct and indirect interactions with leukocytes, in particular neutrophils, are of outmost importance. Future research will further characterize the detailed, spatio-temporal role of platelets in the pathogenesis of distinct tissue- and stimulus-specific inflammatory situations. Furthermore, platelets may also be involved in the resolution of acute inflammation, a field of research of growing importance. A more detailed understanding of the underlying molecular mechanisms will be the key to the development of targeted therapeutic approaches and interventions to improve the treatment of patients suffering from inflammatory diseases.

## Author contributions

All authors listed have made a substantial, direct and intellectual contribution to the work, and approved it for publication.

### Conflict of interest statement

The authors declare that the research was conducted in the absence of any commercial or financial relationships that could be construed as a potential conflict of interest.
